# Sociocognitive factors determining compliance with standard precautions by nursing professionals during the COVID-19 pandemic

**DOI:** 10.1590/0034-7167-2023-0301

**Published:** 2024-09-20

**Authors:** Andressa Silva Torres dos Santos, Ana Cristina de Oliveira e Silva, Fernanda Maria Vieira Pereira-Ávila, Hemílio Fernandes Campos Coêlho, Laelson Rochelle Milanês Sousa, Renata Karina Reis, Elucir Gir

**Affiliations:** I Universidade de São Paulo. Ribeirão Preto, São Paulo, Brazil; II Universidade Federal da Paraíba. João Pessoa, Paraíba, Brazil; III Universidade Federal Fluminense. Rio das Ostras, Rio de Janeiro, Brazil

**Keywords:** Universal Precautions, Nurse Practitioners, Nursing, COVID-19, Pandemics., Precauciones Universales, Enfermeras Practicantes, Enfermería, COVID-19, Pandemias.

## Abstract

**Objectives:**

to assess the socio-cognitive factors determining adherence to standard precautions by nursing professionals in care practice during the COVID-19 pandemic in Brazil.

**Methods:**

an analytical cross-sectional study, carried out with 9,039 nursing professionals in Brazil, using an electronic form containing participant sociodemographic, training and work variables, and the Brazilian version of the Standard Precautions Questionnaire. Descriptive and inferential statistics were used using the statistical software R.

**Results:**

participants recognize standard precautions as effective measures to reduce infections and report intention to perform them. Training regarding standard precautions was evidenced as a facilitator of adherence (4.72; SD: 0.73), and problems related to materials (3.78; SD: 1.45) were a hindrance.

**Conclusions:**

among the determining factors, facilitating organization presented the highest score, followed by intention to perform. Facilitating and hindering factor identification makes it possible to develop intervention strategies to strengthen patient safety and reduce occupational risks among professionals.

## RESUMEN

**Objetivos:**

evaluar los factores sociocognitivos que determinan la adherencia a las precauciones estándar por parte de los profesionales de enfermería en la práctica del cuidado durante la pandemia de COVID-19 en Brasil.

**Métodos:**

estudio analítico transversal, realizado con 9.039 profesionales de enfermería en Brasil, utilizando un formulario electrónico que contiene variables sociodemográficas, de formación y laborales de los participantes, y la versión brasileña del *Standard Precautions Questionnaire*. Se utilizó estadística descriptiva e inferencial mediante el software estadístico R.

**Resultados:**

los participantes reconocen las precauciones estándar como medidas efectivas para reducir las infecciones e informan su intención de seguirlas. La capacitación en precauciones estándar se evidenció como facilitadora de la adherencia (4,72; DE: 0,73), y los problemas relacionados con los materiales (3,78; DE: 1,45) fueron un obstáculo.

**Conclusiones:**

entre los factores determinantes, la organización presentó el puntaje más alto, seguido de la intención de seguir. La identificación de factores facilitadores y obstaculizadores permite desarrollar estrategias de intervención para fortalecer la seguridad del paciente y reducir los riesgos laborales entre los profesionales.

## INTRODUCTION

Standard precautions (SP) consist of preventive measures against infection, which must be followed by healthcare professionals, in any environment, considering that all patients are potentially infected by a pathogen that can be widely disseminated. They include using personal protective equipment (PPE) (cap, protective glasses or face shield, mask, gloves and protective apron), due to the risk of exposure to blood, body fluids, excretions and secretions, in addition to hand hygiene, correct handling and disposal of sharps^(1)^.

In light of the coronavirus disease 2019 (COVID-19) pandemic, among the prevention measures, the relevance of following SP was highlighted, especially in the absence of a confirmed diagnosis of patients, aiming to reduce COVID-19 infection in this scenario, in addition to other occupational risks and minimize cross-transmission of infectious diseases^(2)^.

Studies that investigated adherence to SP showed an insufficient rate among healthcare professionals, with the majority being below what was desired, with rates varying from medium to low, in addition to a high rate of inappropriate use of PPE^(3-7)^.

It is worth highlighting that socio-cognitive determinants encompass social and cognitive factors, which influence human behavior and decision-making, in this case, of nursing professionals. Therefore, socio-cognitive determinants are fundamental to understanding human behavior and, thus, implementing strategies that enable changes. For instance, studies^(8)^ indicate that socio-cognitive factors, such as knowledge, motivation, intention, expectations and perceptions, influence behavior related to hand hygiene. Adherence to SP can be positively influenced by attitudes and behaviors related to the control and prevention of infections, through knowledge and social factors. Therefore, individual and organizational factors can play a relevant role in behavioral intentions regarding this practice^(9-11)^.

Thus, adherence to SP can be hampered by numerous factors: individual factors, such as awareness, risk perception, perception of the effectiveness of protective measures, beliefs and values, knowledge, subjectivity, interpersonal relationships; work-related factors such as workload, work obstacles; and organizational factors, such as availability and quality of PPE, physical structure, supervision, management actions^(3,5,12-13)^.

Among the main reasons, the quantitative and qualitative inadequacy of PPE stands out, while the lack of awareness and deficiencies in training, including personal beliefs, can also be obstacles, as they directly impact personal perception of risk and, consequently, the extent of protection adopted and risk behavior assumed. Furthermore, the lack of continuing education, inadequate related working conditions, mainly excessive working hours, reduced teams and intense work pace are factors that require improvement with the aim of a more favorable environment for adhering to SP. It is worth remembering that, as a consequence of low adherence to SP, work accidents are recorded due to exposure to potentially contaminated biological material and workers becoming ill^(14)^.

In relation to healthcare professionals, it is necessary to highlight nursing professionals, who were on the front line in the fight against COVID-19, given their high representation in the health sector, approximately 59% of the global health workforce^(15)^. Therefore, they were directly impacted by the pandemic and the factors that make it difficult to adhere to SP, recording a high number of confirmed cases and deaths.

Therefore, few studies focus on socio-cognitive determinants, the factors involved and nursing professionals’ behavior. Therefore, it is important to better recognize and assess these determinants through the application of the scale proposed in the present study, aiming to identify the facilitating and hindering factors involved with non-adherence to SP as a preventive measure in patient care, thus enabling the determination of new strategies that promote the reorganization of the work environment and encourage a safety culture, aiming at professional and patient safety.

## OBJECTIVES

To assess the socio-cognitive factors determining SP adherence by nursing professionals in care practice during the COVID-19 pandemic in Brazil.

## METHODS

### Ethical aspects

The project was approved by the Research Ethics Committee (REC) of the *Escola de Enfermagem de Ribeirão Preto, Universidade de São Paulo*.

The scale was used with the authorization of its author, who is also part of this investigation. Data were collected after approval by the REC and upon acceptance of the Informed Consent Form (ICF), available online next to the form, by selecting the option “I have read and agree to participate in the research”.

### Study design and location

This is a cross-sectional and analytical study, carried out through an online survey with nursing professionals from all regions of Brazil. The research followed the recommendations of STrengthening the Reporting of OBservational studies in Epidemiology for RDS studies (STROBE-RDS) and was guided by the Checklist for Reporting Results of Internet E-Surveys (CHERRIES).

### Sample and inclusion criteria

Nursing professionals aged 18 or older, with internet access and who worked in direct assistance to patients, whether or not affected by COVID-19, in the different health care settings, in Brazilian territory, during the COVID-19 pandemic, at least in the last six months prior to the beginning of collection, were included. Professionals who did not respond to the research instrument completely were excluded.

### Data collection

Research participants were recruited using an adaptation of the Respondent Driven Sampling (RDS) method to the virtual environment. Thus, 47 research leaders were selected by the research team, at least one from each state in Brazil, responsible for nominating ten recruiters. Each recruiter nominated ten research participants and they nominated other potential participants, and so on. Leaders and recruiters underwent online training on how to conduct an online survey in the context of the pandemic and on the questionnaire to be administered. A total of 280 collectors were trained in 45 sessions.

A pilot study was carried out with 47 respondents, who sent feedback or comments about the survey. The suggested changes were considered and small adaptations of terminology.

Data collection took place from October 1 to December 31, 2020, using an electronic form, created by the research team and assessed in terms of face and content by 15 experts on the subject. It was made available through a link to SurveyMonkey^®^, the software in which the completed instruments were hosted.

### Data collection instruments and study variables

The electronic form consisted of two parts. The first contained objective questions with independent variables regarding sociodemographic, training and work characteristics.

The second part of the form was composed of the Brazilian version of the Standard Precautions Questionnaire (SPQ-PB)^(16)^, which assesses the socio-cognitive factors determining SP adherence in hospital settings, covering attitudes, behaviors, individual and organizational limitations and constraints. It has 24 items distributed across seven determining socio-cognitive factors: 1 - Attitude toward standard precautions (items 1 to 3); 2 - Social influence (items 4 to 7); 3 - Facilitating organization (items 8 to 10); 4 - Exemplary behavior (items 11 and 12); 5 - Organizational constraints (items 13 to 16); 6 - Individual constraints (items 17 to 20); 7 - Intention to perform standard precautions (items 21 to 24). The answer options consist of a scale ranging from 1 to 5.

### Data treatment and analysis

The collected data were exported and analyzed by the statistical software R, version 4.1.1, using descriptive statistics, with absolute and relative frequency measures for all categorical variables, and central tendency (mean) and dispersion (standard deviation) for all continuous variables.

The overall score, by item and by socio-cognitive factors determining the scale were calculated through the average of the answers obtained on a Likert scale from 1 to 5. The determining socio-cognitive factors assessed consist of the seven factors of the scale. To compare the means between the groups, the scores of SPQ-PB domains and professional categories were used, applying the Mann-Whitney test. The analyzes considered a significance level of 5% (α=0.05).

## RESULTS

Nursing professionals 9,039 (100%) participated in the study (Table 1), the majority of whom were female (7,634; 84.5%) and aged between 18 and 30 years (3,350; 80%). The median age was 34 years (IQR= 12). Time since graduation was ten years, and the experience in the role was nine years. The majority are from the Northeast region (2,728; 30.2%), followed by the Southeast region (2,524; 27.9%). As for professional performance, the largest portion of participants provided assistance to the general public and with COVID-19 (3,810; 42.2%), were from a public institution (6,949; 76.9%) and from the infirmary sector (2,382; 26.4%).

**Table 1:** Sociodemographic and occupational characteristics of nursing professionals (N=9,039), Brazil, 2020

Participant sociodemographic and occupational characteristics		
Variables	n	%
Sex		
Male	1,405	15.5%
Feale	7,634	84.5%
Region		
Northeast	2,718	30.2%
Southeast	2,524	27.9%
Midwest	1,609	17.8%
North	1,376	15.2%
South	802	8.9%
Professional category		
Nurse	5,890	65.2%
Nursing technician or assistant	3,149	34.8%
Time since graduation		
0 to 5 years	4,295	47.5%
6 to 10 years	2,053	22.7%
11 years and over	2,691	29.8%
Length of professional experience		
0 to 5 years	3,666	40.6%
6 to 10 years	2,280	25.2%
11 years and over	3,093	34.2%
Type of person providing assistance		
With COVID (suspected or confirmed)	1,677	18.6%
General	3,552	39.3%
Both	3,810	42.2%
Type of institution where they work		
Did not answer this item	7,245	80.2%
Private	1,794	19.8%
Type of institution where they work		
Did not answer this item	2,090	23.1%
Public	6,949	76.9%
Sector(s) of activity		
Did not answer this item	8,198	90.7%
Outpatient clinic	841	9.3%
Sector(s) of activity		
Did not answer this item	6,966	77.1%
Intensive Care Unit	2,073	22.9%
Sector(s) of activity		
Did not answer this item	6657	73.6%
Ward	2382	26.4%
Sector(s) of activity		
Did not answer this item	8,435	93.3%
Operating Room	604	6.7%
Sector(s) of activity		
Did not answer this item	7,719	85.4%
Emergency Care Unit	1,320	14.6%
Sector(s) of activity		
Did not answer this item	7,491	82.9%
Emergency Unit	1,548	17.1%
Sector(s) of activity		
Did not answer this item	7,481	82.8%
Basic Health Unit	1,558	17.2%
Sector(s) of activity		
Did not answer this item	8,961	99.1%
Private office	78	0.9%
Sector(s) of activity		
Did not answer this item	8,287	91.7%
Field hospital to assist patients with COVID-19	752	8.3%
Sector(s) of activity		
Did not answer this item	7,821	86.5%
Other	1,218	13.5%
COVID diagnosis (clinical or laboratory)		
No	5,983	66.2%
Yes	3,056	33.8%
Isolation for a period from the family		
Yes	5,879	65.0%
No	3,035	33.6%
Not applicable	125	1.4%
Training or course on COVID-19		
Yes	6,335	70.1%
No	2,704	29.9%
Institution working provided sufficient personal protective equipment		
Yes	6,550	72.5%
No	457	5.1%
In part	2,032	22.5%
Institution working provided good quality personal protective equipment		
Yes	4,808	53.2%
No	973	10.8%
In part	3,258	36.0%

Most participants recognized SP as effective measures to reduce infections, recognizing their importance. Training regarding SP was highlighted by professionals as facilitating adherence, while problems related to the material, followed by a lack of knowledge about SP and a higher workload than usual, were indicated as complicating factors, as shown in Table 2. Participants reported the intention to follow SP even when patients are difficult or there is little time.

**Table 2:** Distribution, percentage, mean and standard deviation according to items from the Brazilian version of the Standard Precautions Questionnaire, answered by nursing professionals (N=9,039), Brazil, 2020

		Answer options						
**Scale item**		**Not effective at all** **n (%)**	**Somewhat effective** **n (%)**	**More or less effective** **n (%)**	**Partially effective** **n (%)**	**Totally effective** **n (%)**	**Mean**	**Standard deviation**
1	*As precauções-padrão são medidas eficazes para reduzir as infecções hospitalares* (Standard precautions are effective in reducing health care infection)	75 (0.8)	9 (0.1)	2,508 (27.7)	174 (1.9)	6,273 (69.4)	4.39	0.95
2	*Se eu seguir as precauções-padrão, protegerei meus pacientes de uma infecção* (If I follow the protocol of standard precautions, I will protect my patients from infection)	60 (0.7)	11 (0.1)	2,190 (24.2)	175 (1.9)	6,603 (73.1)	4.47	0.91
3	*Seguir as medidas de precauções-padrão vai me proteger de uma infecção* (Following the standard precautions protocol will protect me from infection)	^*^	^*^	^*^	^*^	^*^	^*^	^*^
4	*A maioria dos meus colegas de trabalho pensa que é importante seguir às precauções-padrão* (Most of my colleagues think it is important to adhere to standard precautions)	235 (2.6)	42 (0.5)	4,275 (47.3)	177 (2.0)	4,310 (47.7)	3.92	1.09
5	*Corro o risco de receber advertências dos meus superiores, se não seguir às precauções-padrão* (I will be reprimanded by the charge nurse if I do not adhere to standard precautions)	834 (9.2)	66 (0.7)	2,525 (27.9)	152 (1.7)	5,462 (60.4)	4.03	1.32
6	*Corro o risco de receber advertências dos enfermeiros e auxiliares responsáveis pela higienização, se não seguir às precauções-padrão* (I will be reprimanded by infection control link nurses if I do not adhere to standard precautions)	952 (10.5)	80 (0.9)	2,735 (30.3)	158 (1.7)	5,114 (56.6)	3.93	1.36
7	*Corro o risco de receber advertências dos médicos, se não seguir às precauções-padrão* (I will be reprimanded by the physicians if I do not adhere to standard precautions)	2,789 (30.9)	122 (1.3)	2,871 (31.8)	128 (1.4)	3,129 (34.6)	3.08	1.63
8	*Ter material (qualidade, disponibilidade e acessibilidade) em todos os locais de trabalho* (Having equipment available in the health care setting)	136 (1.5)	20 (0.2)	1,268 (14.0)	85 (0.9)	7,530 (83.3)	4.64	0.84
9	*Estar capacitado no que se refere às precauções-padrão* (To be trained in using standard precautions)	66 (0.7)	13 (0.1)	1,066 (11.8)	96 (1.1)	7,798 (86.3)	4.72	0.73
10	*Ter capacitação quanto às precauções-padrão* (To receive reminders about standard precautions)	78 (0.9)	15 (0.2)	1,069 (11.8)	101 (1.1)	7,776 (86.0)	4.71	0.74
11	*Quando o profissional médico tem um comportamento exemplar em relação às precauções-padrão* (The senior physician has exemplary behavior regarding adherence to standard precautions)	618 (6.8)	47 (0.5)	2,518 (27.9)	125 (1.4)	5,731 (63.4)	4.14	1.24
**Scale item**		**Not an obstacle** **n (%)**	**A bit of an obstacle** **n (%)**	**More or less obstacle** **n (%)**	**Partially obstacle** **n (%)**	**An** **obstacle** **n (%)**	**Mean**	**Standard deviation**
12	*Quando meus colegas de trabalho têm um comportamento exemplar em relação às precauções-padrão* (My colleagues have exemplary behavior regarding adherence to standard precautions)	154 (1.7)	34 (0.4)	2,192 (24.3)	153 (1.7)	6,506 (72.0)	4.42	0.98
13	*Situações inesperadas que podem atrapalhar a realização de meu trabalho (urgência, solicitação de colegas, nova tarefa a cumprir)* (The occurrence of unanticipated events that adversely affect my work)	1,998 (22.1)	63 (0.7)	4,610 (51.0)	90 (1.0)	2,278 (25.2)	3.07	1.38
14	*Falta de tempo* (Lack of time)	2,893 (32.0)	56 (0.6)	3,432 (38.0)	72 (0.8)	2,586 (28.6)	2.93	1.56
15	*Carga de trabalho mais elevada que de costume* (Increased worload)	1,863 (20.6)	54 (0.6)	2,887 (31.9)	96 (1.1)	4,139 (45.8)	3.51	1.55
16	*Complexidade das medidas de precauções-padrão* (The complexity of the standard precautions protocol)	3,564 (39.4)	76 (0.8)	3,596 (39.8)	80 (0.9)	1,723 (19.1)	2.59	1.48
17	*Falta de conhecimento sobre as precauções-padrão* (Lack of knowledge about standard precautions)	1,672 (18.5)	52 (0.6)	2,381 (26.3)	58 (0.6)	4,876 (53.9)	3.71	1.55
18	*Rotina, hábitos e equipe de trabalho* (Care team routine)	2,903 (32.1)	57 (0.6)	3,723 (41.2)	95 (1.1)	2,261 (25.0)	2.86	1.51
19	*Crenças pessoais relacionadas às precauções-padrão* (Personal beliefs about standard precautions)	3,725 (41.2)	75 (0.8)	2,768 (30.6)	55 (0.6)	2,416 (26.7)	2.71	1.63
**Scale item**		**Never** **n (%)**	**Rarely** **n (%)**	**Sometimes** **n (%)**	**Often** **n (%)**	**Ever** **n (%)**	**Mean**	**Standard deviation**
20	*Problemas relacionados ao material (qualidade, disponibilidade e acessibilidade)* (Problems related to use of equipment)	1,278 (14.1)	50 (0.6)	2,823 (31.2)	86 (1.0)	4,802 (53.1)	3.78	1.45
21	*Mesmo quando o paciente for difícil* (Even if the patient is difficult)	103 (1.1)	78 (0.9)	353 (3.9)	1,281 (14.2)	7,224 (79.9)	4.71	0.69
22	*Mesmo quando houver pouco tempo* (Even if I am pressed for time)	108 (1.2)	145 (1.6)	657 (7.3)	1,873 (20.7)	6,256 (69.2)	4.55	0.80
23	*Mesmo quando minhas mãos estiverem doloridas ou machucadas* (Even if my hands are damaged or painful)	207 (2.3)	219 (2.4)	728 (8.1)	1,653 (18.3)	6,232 (68.9)	4.49	0.91
24	*Mesmo em uma situação de urgência* (During an emergency situation)	125 (1.4)	208 (2.3)	818 (9.0)	1,979 (21.9)	5,909 (65.4)	4.48	0.86

* *Missing.*

The SPQ scale presented an overall mean of 3.91 (SD= 0.48). Regarding the scale domains, the facilitating organization factor presented the highest score (4.69; SD: 0.68), followed by intention to perform standard precautions (4.56; SD: 0.70) (Table 3).

**Table 3:** Score of socio-cognitive determinants referring to the factors of the Brazilian version of the Standard Precautions Questionnaire of nursing professionals (N=9,039), Brazil, 2020

Factors	Mean	Standard deviation
*Atitudes* (Attitude toward standard precautions)	4.19	0.81
*Influência social* (Social influence)	3.74	1.06
*Organização* (Facilitating organization)	4.69	0.68
*Comportamento interpessoal* (Exemplary behavior)	4.28	1.01
*Restrições organizacionais* (Organizational constraints)	3.03	1.19
*Restrições individuais* (Individual constraints)	3.27	1.16
*Intenção de seguir as precauções-padrão* (Intention to perform standard precautions)	4.56	0.70

In the analysis of SPQ-PB factor scores according to professional category (Table 4), nurses had a significant effect on facilitating organization (nurse: 4.72/nursing assistant or technician: 4.63; p<0.01), individual constraints (nurse: 3.35/nursing assistant or technician: 3.11; p<0.01) and intention to perform standard precautions (nurse: 4.58/nursing assistant or technician: 4.52; p<0.01) scores. Meanwhile, technicians and assistants scored higher in other factors, such as attitude toward standard precautions (nursing assistant or technician: 4.24/nurse: 4.17; p<0.01), social influence (nursing assistant or technician: 3.99/nurse: 3.60; p<0.01) and exemplary behavior (nursing assistant or technician: 4.39/nurse: 4.22; p<0.01). For the organizational constraints factor, there were no significant differences between the categories (p = 0.250).

**Table 4:** Score of socio-cognitive determinants referring to the factors of the Brazilian version of the Standard Precautions Questionnaire by professional category of nursing professionals (N=9,039), Brazil, 2020

Factors	Professional category		
	Nurse	Nursing assistant or technician	*p* value^*^
*Atitudes* (Attitude toward standard precautions)	4.17	4.24	< 0.01
*Influência social* (Social influence)	3.60	3.99	< 0.01
*Organização* (Facilitating organization)	4.72	4.63	< 0.01
*Comportamento interpessoal* (Exemplary behavior)	4.22	4.39	< 0.01
*Restrições organizacionais* (Organizational constraints)	3.04	3.00	0.250
*Restrições individuais* (Individual constraints)	3.35	3.11	< 0.01
*Intenção de seguir as precauções-padrão* (Intention to perform standard precautions)	4.58	4.52	< 0.01

* *Mann-Whitney test.*

## DISCUSSION

The present study assessed the socio-cognitive factors determining SP adherence by nursing professionals in care practice during the COVID-19 pandemic in Brazil. Participants recognized SP as effective measures to reduce infections and reported their intention to perform them.

The perception of effectiveness, knowledge of preventive measures as well as intention to perform them are fundamental, as this is an individual factor that directly interferes with the process of whether or not health workers adhere to SP^(12)^, as step that imply personal perception of risk and, consequently, the extent of protection adopted and risk behavior assumed. Therefore, professionals who recognize their work environment as low or medium risk are more likely to have an accident, when compared to those who assess the risk as high, because adherence to PPE is closely related to the perception that professionals have about the risks to which they are exposed^(17)^.

A study carried out in midwestern Brazil found that the nursing team’s knowledge about SP was lower than recommended. Furthermore, it showed that individual factors, related to work, including the perception of risk and obstacles to following SP, in addition to organizational factors, such as training and availability of PPE, impact SP adherence^(18)^. Such data converge with the findings of this research, in which, in the domains of organizational and individual constraints, problems related to the material, including quality, availability and accessibility of PPE, were indicated as complicating factors, followed by the lack of knowledge about SP, mentioned previously, and a higher workload.

Unsatisfactory knowledge can impact low adherence rates and may be correlated with gaps in professional training, in addition to self-confidence resulting from years of professional experience, gap between knowledge and practice, and out-of-date current research, hampering solid knowledge bases for infection prevention and control. Furthermore, long working hours can trigger stress and fatigue, which can affect professionals’ cognition processes, with consequent harm to the application of safety measures^(3,19)^, such as SP.

With regard to the facilitating organization domain, training in SP was highlighted as a facilitator of adherence. Thus, the need for training is highlighted, through health education activities with a focus on SP adherence and its intervening factors, as it is a complex, dynamic, multifaceted topic of organizational responsibility, which enables professionals to raise awareness and awareness regarding the relevance of adhering to SP^(18-19)^. It is noteworthy that professionals show greater adherence immediately after training carried out by health institutions, however, measures are discontinued over time. Therefore, the relevance of continued and permanent educational strategies is observed, in addition to constant training for action effectiveness^(3,19)^.

Among the main reasons for low SP adherence and contact, the review highlighted individual factors, deficiencies in training, organizational structure, problems related to unit management and working conditions^(3)^. Therefore, institutional management is essential for the team’s participation in these activities and training to occur continuously, through a care environment favorable to adherence, with PPE in sufficient quality and quantity, easily accessible, to facilitate and encourage its use, in addition to an institutional safety climate^(18)^, provided by a physical structure and quality supervision, combined with standardization and socialization of routines^(12)^. An unsafe environment implies a reduction in professionals’ motivation and interest, predisposing them to errors and injuries^(20)^.

With regard to the work environment/social influence, they recognize that they run the risk of receiving warnings from their superiors if they do not follow SP, which demonstrates management’s concern regarding the topic and its implications for professionals and patients. This reflects a positive institutional perception, as long as a favorable care environment is provided for the application of these measures, such as physical structure and quality materials, as mentioned previously.

Constant coexistence with exposure to biological material, which impacts the lack of fear of contamination, and the belief that nothing will happen, increasing workers’ feeling of self-confidence, favor the reduction of risk perception by professionals, one of the most complex (individual) factors, influencing professionals’ behavior and hindering decision-making for SP adoption^(12)^. Therefore, having a co-worker adopting preventive measures correctly favors risk environment perception and greater SP adherence, whereas, in environments where interpersonal relationships are not healthy, it impacts the lack of motivation for use. Therefore, co-workers can influence positively or negatively^(20)^.

Therefore, adherence to PPE is determined by the context experienced, in the work environment, and by individual values and beliefs, but the decision about use in general is individual. Individual factors, such as discomfort, inconvenience, carelessness, forgetfulness, lack of habit and disbelief, can be greatly aggravated by institutional/organizational, such as precarious infrastructure, lack of sufficient and quality PPE, lack of knowledge due to the lack of permanent education and work overload, which imply physical and mental exhaustion, impacting cognition^(20)^. Therefore, recognizing individual and institutional factors is of utmost importance, aiming to provide bases that minimize barriers, weaken the perception of susceptibility and severity to risk and reduce SP adherence, favoring facilitators.

A study that applied the SPQ-PB with 300 healthcare professionals showed that nurses had a significant effect on intention (4.77; p=0.000) and individual constraints (3.52; p=0.041)^(16)^ scores, similar to present research.

### Study limitations

The research presents as a limitation the possibility of an overrepresentation of professionals with skills in relation to the use of computers and social networks, given their online development. However, given the high number of participants, such limitations did not significantly interfere with the results.

### Contributions to nursing, health, or public policy

The results can contribute to a greater understanding of the determining factors in SP adherence and, thus, enable new intervention strategies to reduce hindering factors and promote facilitators in these professionals’ work practice, including the prioritization of this topic in training programs, elaboration and/or reformulation of public policies and institutional protocols, thus aiming to reduce cross-infections and occupational risks caused by low adherence or erroneous use of SP, with consequent institutional losses.

## CONCLUSIONS

Among the determining factors in SP adherence by nursing professionals in care practice during the COVID-19 pandemic in Brazil, the facilitating organization factor presented the highest score, therefore, the greatest impact on adherence, followed by intention to perform standard precautions. Furthermore, participants recognized SP as effective measures to reduce infections and highlighted training as a facilitator of SP adherence and problems related to the material as the main obstacle.

In view of these findings, it was possible to develop intervention strategies, training, training and continuing education that focus on the main factors determining SP adherence, in addition to targeting strategies to improve adherence to them, to thus strengthen patient safety, reduce exposure and occupational risks among healthcare professionals as well as the occurrence of occupational accidents.
