# Supplementary material for: Sociocognitive factors determining compliance with standard precautions by nursing professionals during the COVID-19 pandemic
Source: Rev Bras Enferm. 2024 Sep 20;77(4):e20230301. doi: 10.1590/0034-7167-2023-0301 (PMC11419678; doi:10.1590/0034-7167-2023-0301)
Supplement: Translation

# Translation: Fatores sociocognitivos determinantes na adesão às precauções padrão pelos profissionais de enfermagem na pandemia de COVID-19

**DOI:** 10.1590/0034-7167-2023-0301pt

**Authors:** Andressa Silva Torres dos Santos, Ana Cristina de Oliveira e Silva, Fernanda Maria Vieira Pereira-Ávila, Hemílio Fernandes Campos Coêlho, Laelson Rochelle Milanês Sousa, Renata Karina Reis, Elucir Gir

**Affiliations:** I Universidade de São Paulo. Ribeirão Preto, São Paulo, Brasil; II Universidade Federal da Paraíba. João Pessoa, Paraíba, Brasil; III Universidade Federal Fluminense. Rio das Ostras, Rio de Janeiro, Brasil

**Keywords:** Precauções Universais, Profissionais de Enfermagem, Enfermagem, COVID-19, Pandemias

## Abstract

**Objetivos:**

avaliar os fatores sociocognitivos determinantes na adesão às precauções padrão pelos profissionais de enfermagem na prática assistencial na pandemia de COVID-19 no Brasil.

**Métodos:**

estudo transversal analítico, realizado com 9.039 profissionais de enfermagem do Brasil, através de um formulário eletrônico contendo variáveis sociodemográficas, de formação e laborais dos participantes, e a versão brasileira do *Standard Precautions Questionnaire*. Utilizou-se estatística descritiva e inferencial pelo *software* estatístico R.

**Resultados:**

os participantes reconhecem precauções padrão como medidas eficazes para reduzir infecções e relataram intenção de segui-las. A capacitação quanto às precauções padrão foi evidenciada como facilitador da adesão (4,72; DP: 0,73), e problemas relacionados a materiais (3,78; DP: 1,45) foi um dificultador.

**Conclusões:**

entre os fatores determinantes, a organização apresentou maior pontuação, seguida da intenção de seguir. A identificação dos fatores facilitadores e dificultadores possibilita desenvolver estratégias interventivas visando fortalecer a segurança do paciente e diminuir os riscos ocupacionais entre profissionais.

## INTRODUÇÃO

As precauções padrão (PP) consistem em medidas preventivas de infecção que devem ser seguidas pelos profissionais de saúde, em qualquer ambiente, considerando que todos os pacientes estão potencialmente infectados por patógeno que pode ser amplamente disseminado. Incluem o uso de equipamento de proteção individual (EPI) (gorro, óculos de proteção ou protetor facial, máscara, luvas e avental de proteção), diante do risco de exposição a sangue, fluídos corporais, excreções e secreções, além da higienização das mãos, manejo e descarte correto de materiais perfurocortantes^(1)^.

Diante da pandemia do *coronavirus disease* 2019 (COVID-19), entre as medidas de prevenção, destacou-se a relevância de seguir as PP, principalmente diante da ausência de diagnóstico confirmado do paciente, visando reduzir a infecção pela COVID-19 neste cenário, além de outros riscos ocupacionais e minimizar transmissão cruzada de doenças infectocontagiosas^(2)^.

Estudos que investigaram a adesão às PP evidenciaram uma taxa insuficiente entre profissionais de saúde, estando, de forma majoritária, aquém do desejado, com índices variando de médios a baixos, para além de um índice elevado do uso inadequado dos EPI^(3-7)^.

Vale destacar que os determinantes sociocognitivos abarcam os fatores sociais e cognitivos, que influenciam o comportamento humano e a tomada de decisão, nesse caso, dos profissionais de enfermagem. Portanto, os determinantes sociocognitivos são fundamentais para entender o comportamento humano e, assim, implementar estratégias que possibilitem mudanças. Por exemplo, estudos^(8)^ indicam que os fatores sociocognitivos, como conhecimento, motivação, intenção, expectativas e percepções, exercem influência sobre o comportamento relativo à higiene das mãos. A adesão às PP pode ser influenciada de forma positiva nas atitudes e nos comportamentos relativos ao controle e à prevenção de infecções, pelo conhecimento e fatores sociais. Logo, fatores individuais e organizacionais podem desempenhar um papel relevante nas intenções comportamentais em relação a essa prática^(9-11)^.

Assim, a adesão às PP pode ser dificultada por inúmeros fatores: fatores individuais, como conscientização, percepção do risco, percepção da eficácia das medidas de proteção, crenças e valores, conhecimento, subjetividade, relações interpessoais; fatores relacionados ao trabalho, como carga de trabalho, obstáculos do trabalho; e fatores organizacionais, como disponibilidade e qualidade dos EPI, estrutura física, supervisão, ações gerenciais^(3,5,12-13)^.

Entre os principais motivos, ressalta-se a inadequação quanti-qualitativa de EPI, enquanto a falta de conscientização e deficiências na formação, incluindo crenças pessoais, também podem ser obstáculos, visto que impactam diretamente a percepção pessoal do risco e, consequentemente, a extensão da proteção adotada e o comportamento de risco assumido. Além disso, a falta de educação permanente, inadequadas condições de trabalho relacionadas, principalmente a jornada excessiva, equipes reduzidas e ritmo intenso de trabalho são fatores que carecem de melhorias com o objetivo de um ambiente mais favorável para adesão às PP. Vale lembrar que, como consequências da baixa adesão às PP, registram-se acidentes de trabalho diante da exposição a material biológico potencialmente contaminado e adoecimento de trabalhadores^(14)^.

Referente aos profissionais de saúde, é preciso destacar os profissionais de enfermagem, que estiveram na linha de frente do enfrentamento da COVID-19, diante da sua alta representatividade no setor da saúde, aproximadamente 59% da força de trabalho em saúde mundial^(15)^. Logo, foram impactados diretamente pela pandemia e pelos fatores que dificultam a adesão às PP, registrando um número elevado de casos confirmados e óbitos.

Desse modo, poucos estudos se debruçam sobre os determinantes sociocognitivos, seus fatores envolvidos e o comportamento dos profissionais de enfermagem. Portanto, torna-se importante reconhecer e avaliar melhor esses determinantes por meio da aplicação da escala proposta no presente estudo, visando identificar os fatores facilitadores e dificultadores envolvidos com a não adesão à PP como medida preventiva no atendimento ao paciente, para, assim, possibilitar a determinação de novas estratégias que promovam a reorganização do ambiente laboral e incentivem a cultura de segurança, visando à segurança do profissional e do paciente.

## OBJETIVOS

Avaliar os fatores sociocognitivos determinantes na adesão às PP pelos profissionais de enfermagem na prática assistencial na pandemia de COVID-19 no Brasil.

## MÉTODOS

### Aspectos éticos

O projeto foi aprovado pelo Comitê de Ética em Pesquisa (CEP) da Escola de Enfermagem de Ribeirão Preto da Universidade de São Paulo.

A utilização da escala aconteceu mediante autorização da autora da mesma, que integra também a presente investigação. Os dados foram coletados após a aprovação do CEP e mediante aceite do Termo de Consentimento Livre Esclarecido (TCLE), disponibilizado *online* junto ao formulário, através da seleção da opção “Li e concordo participar da pesquisa”.

### Desenho e local do estudo

Estudo transversal e analítico, realizado por meio de inquérito *online* com profissionais de enfermagem de todas as regiões do Brasil. A pesquisa seguiu as recomendações do *STrengthening the Reporting of Observational studies in Epidemiology for RDS studies* (STROBE-RDS) e norteou-se pelo *Checklist for Reporting Results of Internet E-Surveys* (CHERRIES).

### Amostra e critérios de inclusão

Foram incluídos profissionais de enfermagem com idade igual ou superior a 18 anos, acesso à internet e que atuaram na assistência direta aos pacientes, acometidos ou não pela COVID-19, nos diferentes cenários de atenção à saúde, em território brasileiro, durante a pandemia de COVID-19, pelo menos nos últimos seis meses que antecederam o início da coleta. Foram excluídos profissionais que não responderam ao instrumento de pesquisa de forma completa.

### Coleta de dados

Os participantes da pesquisa foram recrutados utilizando uma adaptação do método *Respondent Driven Sampling* (RDS) ao ambiente virtual. Assim, 47 líderes de pesquisa foram selecionados pela equipe de pesquisa, sendo pelo menos um de cada estado do Brasil, responsáveis por indicar dez recrutadores. Cada recrutador indicou dez participantes para pesquisa, e esses indicaram outros possíveis participante, e assim sucessivamente. Os líderes e recrutadores passaram por um treinamento *online* sobre como realizar pesquisa *online* no contexto da pandemia e sobre o questionário a ser aplicado. Obtiveram-se 280 coletadores treinados em 45 sessões.

Estudo piloto foi realizado com 47 respondentes, que enviaram *feedback* ou comentários sobre a pesquisa. As mudanças sugeridas foram consideradas, realizando-se pequenas adaptações de terminologia.

A coleta dos dados ocorreu no período de 01 de outubro a 31 de dezembro de 2020, através de um formulário eletrônico, construído pela equipe de pesquisa e avaliado quanto à face e ao conteúdo por 15 especialistas na temática. O mesmo foi disponibilizado por meio de um *link* do *SurveyMonkey* ^®^, *software* no qual os instrumentos preenchidos foram hospedados.

### Instrumentos de coleta de dados e variáveis do estudo

O formulário eletrônico foi composto por duas partes. A primeira continha questões objetivas com variáveis independentes quanto às características sociodemográficas, de formação e laborais.

A segunda parte do formulário foi composta pela versão brasileira do *Standard Precautions Questionnaire* (SPQ-PB)^(16)^, que avalia os fatores sociocognitivos determinantes na adesão às PP no ambiente hospitalar, abrangendo atitudes, comportamentos, limitações e restrições pessoais e organizacionais. O mesmo conta com 24 itens, distribuídos em sete fatores sociocognitivos determinantes: 1 - Atitude (itens 1 ao 3); 2 - Influência social (itens 4 ao 7); 3 - Organização (itens 8 ao 10); 4 - Comportamento interpessoal (itens 11 e 12); 5 - Restrições organizacionais (itens 13 ao 16); 6 - Restrições individuais (itens 17 ao 20); 7 - Intenção de seguir as precauções-padrão (itens 21 ao 24). As opções de resposta consistem em uma escala que varia de 1 a 5.

### Tratamento e análise dos dados

Os dados coletados foram exportados e analisados pelo *software* estatístico R, versão 4.1.1, utilizando estatística descritiva, com medidas de frequência absoluta e relativa para todas as variáveis categóricas, e tendência central (média) e dispersão (desvio padrão) para todas as variáveis contínuas.

O escore geral, por item e por fatores sociocognitivos determinantes da escala, foi calculado através da média das respostas obtidas em uma escala *Likert* de 1 a 5. Os fatores sociocognitivos determinantes avaliados consistem nos sete fatores da escala. Para comparação das médias entre os grupos, utilizaram-se os escores dos domínios do SPQ-PB e as categorias profissionais, aplicando o Teste de *Mann-Whitney*. As análises consideraram nível de significância de 5% (α=0,05).

## RESULTADOS

Participaram do estudo 9.039 (100%) profissionais de enfermagem (Tabela 1), sendo a maioria do sexo feminino (7.634; 84,5%) e com idade entre 18 e 30 anos (3.350; 80%). A idade mediana foi de 34 anos (IQR= 12). O tempo de formado foi de dez anos, e de tempo de experiência na função, nove anos. A maioria é da região Nordeste (2.728; 30,2%), seguida da região Sudeste (2.524; 27,9%). Quanto à atuação profissional, a maior parcela dos participantes prestou assistência ao público geral e com COVID-19 (3.810; 42,2%), era de instituição pública (6.949; 76,9%) e do setor da enfermaria (2.382; 26,4%).

**Tabela 1:** Características sociodemográficas e ocupacionais dos profissionais de enfermagem (N=9.039), Brasil, 2020

Características sociodemográficas e ocupacionais dos participantes		
Variáveis	n	%
Sexo		
Masculino	1.405	15,5%
Feminino	7.634	84,5%
Região		
Nordeste	2.718	30,2%
Sudeste	2.524	27,9%
Centro-Oeste	1.609	17,8%
Norte	1.376	15,2%
Sul	802	8,9%
Categoria profissional		
Enfermeiro	5.890	65,2%
Técnico de enfermagem ou auxiliar	3.149	34,8%
Tempo de formação		
0 a 5 anos	4.295	47,5%
6 a 10 anos	2.053	22,7%
11 anos ou mais	2.691	29,8%
Tempo de experiência profissional		
0 a 5 anos	3.666	40,6%
6 a 10 anos	2.280	25,2%
11 anos ou mais	3.093	34,2%
Tipo de pessoa que presta assistência		
Com COVID (suspeita ou confirmado)	1.677	18,6%
Em geral	3,552	39,3%
Ambos	3.810	42,2%
Tipo de instituição onde trabalha		
Não respondeu este item	7.245	80,2%
Privada	1.794	19,8%
Tipo de instituição onde trabalha		
Não respondeu este item	2.090	23,1%
Pública	6.949	76,9%
Setor(es) de atuação		
Não respondeu este item	8.198	90,7%
Ambulatório	841	9,3%
Setor(es) de atuação		
Não respondeu este item	6.966	77,1%
Unidade de Terapia Intensiva	2.073	22,9%
Setor(es) de atuação		
Não respondeu este item	6657	73,6%
Enfermaria	2382	26,4%
Setor(es) de atuação		
Não respondeu este item	8.435	93,3%
Centro Cirúrgico	604	6,7%
Setor (es) de atuação		
Não respondeu este item	7.719	85,4%
Unidade de Pronto Atendimento	1.320	14,6%
Setor(es) de atuação		
Não respondeu este item	7.491	82,9%
Unidade de Emergência	1.548	17,1%
Setor(es) de atuação		
Não respondeu este item	7.481	82,8%
Unidade Básica de Saúde	1.558	17,2%
Setor(es) de atuação		
Não respondeu este item	8.961	99,1%
Consultório particular	78	0,9%
Setor(es) de atuação		
Não respondeu este item	8.287	91,7%
Hospital de campanha para assistência a pacientes com COVID-19	752	8,3%
Setor(es) de atuação		
Não respondeu este item	7.821	86,5%
Outro	1.218	13,5%
Diagnóstico de COVID (clínico ou laboratorial)		
Não	5.983	66,2%
Sim	3.056	33,8%
Isolamento por um período da família		
Sim	5.879	65,0%
Não	3.035	33,6%
Não se aplica	125	1,4%
Capacitação ou curso sobre a COVID-19		
Sim	6.335	70,1%
Não	2.704	29,9%
Instituição que trabalha forneceu equipamento de proteção individual suficiente		
Sim	6.550	72,5%
Não	457	5,1%
Em parte	2.032	22,5%
Instituição que trabalha forneceu equipamento de proteção individual de boa qualidade		
Sim	4.808	53,2%
Não	973	10,8%
Em parte	3.258	36,0%

A maioria dos participantes reconheceu as PP como medidas eficazes para reduzir as infecções, reconhecendo sua importância. A capacitação quanto às PP foi evidenciada pelos profissionais como facilitadora da adesão, enquanto que problemas relacionados ao material, seguidos da falta de conhecimento sobre as PP e carga de trabalho mais elevada que de costume, foram indicados como fatores dificultadores, conforme Tabela 2. Os participantes relataram intenção de seguir as PP mesmo quando o paciente for difícil ou houver pouco tempo.

**Tabela 2:** Distribuição, percentual, média e desvio padrão segundo itens da versão brasileira do *Standard Precautions Questionnaire*, respondidos pelos profissionais de enfermagem (N=9.039), Brasil, 2020

		Opções de respostas						
**Item da escala**		**Nada eficaz** **n (%)**	**Um pouco eficaz** **n (%)**	**Mais ou menos eficaz** **n (%)**	**Parcialmente eficaz** **n (%)**	**Totalmente eficaz** **n (%)**	**Média**	**Desvio padrão**
1	As precauções-padrão são medidas eficazes para reduzir as infecções hospitalares	75 (0,8)	9 (0,1)	2.508 (27,7)	174 (1,9)	6.273 (69,4)	4,39	0,95
2	Se eu seguir as precauções-padrão, protegerei meus pacientes de uma infecção	60 (0,7)	11 (0,1)	2.190 (24,2)	175 (1,9)	6.603 (73,1)	4,47	0,91
3	Seguir as medidas de precauções-padrão vai me proteger de uma infecção	^*^	^*^	^*^	^*^	^*^	^*^	^*^
4	A maioria dos meus colegas de trabalho pensa que é importante seguir às precauções-padrão	235 (2,6)	42 (0,5)	4.275 (47,3)	177 (2,0)	4.310 (47,7)	3,92	1,09
5	Corro o risco de receber advertências dos meus superiores, se não seguir às precauções-padrão	834 (9,2)	66 (0,7)	2.525 (27,9)	152 (1,7)	5.462 (60,4)	4,03	1,32
6	Corro o risco de receber advertências dos enfermeiros e auxiliares responsáveis pela higienização, se não seguir às precauções-padrão	952 (10,5)	80 (0,9)	2.735 (30,3)	158 (1,7)	5.114 (56,6)	3,93	1,36
7	Corro o risco de receber advertências dos médicos, se não seguir às precauções-padrão	2.789 (30,9)	122 (1,3)	2.871 (31,8)	128 (1,4)	3.129 (34,6)	3,08	1,63
8	Ter material (qualidade, disponibilidade e acessibilidade) em todos os locais de trabalho	136 (1,5)	20 (0,2)	1.268 (14,0)	85 (0,9)	7.530 (83,3)	4,64	0,84
9	Estar capacitado no que se refere às precauções-padrão	66 (0,7)	13 (0,1)	1.066 (11,8)	96 (1,1)	7.798 (86,3)	4,72	0,73
10	Ter capacitação quanto às precauções-padrão	78 (0,9)	15 (0,2)	1.069 (11,8)	101 (1,1)	7.776 (86,0)	4,71	0,74
11	Quando o profissional médico tem um comportamento exemplar em relação às precauções-padrão	618 (6,8)	47 (0,5)	2.518 (27,9)	125 (1,4)	5.731 (63,4)	4,14	1,24
**Item da escala**		**Não é obstáculo** **n (%)**	**Um pouco de obstáculo** **n (%)**	**Mais ou menos obstáculo** **n (%)**	**Parcialmente obstáculo** **n (%)**	**É um obstáculo** **n (%)**	**Média**	**Desvio padrão**
12	Quando meus colegas de trabalho têm um comportamento exemplar em relação às precauções-padrão	154 (1,7)	34 (0,4)	2.192 (24,3)	153 (1,7)	6.506 (72,0)	4,42	0,98
13	Situações inesperadas que podem atrapalhar a realização de meu trabalho (urgência, solicitação de colegas, nova tarefa a cumprir)	1.998 (22,1)	63 (0,7)	4.610 (51,0)	90 (1,0)	2.278 (25,2)	3,07	1,38
14	Falta de tempo	2.893 (32,0)	56 (0,6)	3.432 (38,0)	72 (0,8)	2.586 (28,6)	2,93	1,56
**Item da escala**		**Não é obstáculo** **n (%)**	**Um pouco de obstáculo** **n (%)**	**Mais ou menos obstáculo** **n (%)**	**Parcialmente obstáculo** **n (%)**	**É um obstáculo** **n (%)**	**Média**	**Desvio padrão**
15	Carga de trabalho mais elevada que de costume	1.863 (20,6)	54 (0,6)	2.887 (31,9)	96 (1,1)	4.139 (45,8)	3,51	1,55
16	Complexidade das medidas de precauções-padrão	3.564 (39,4)	76 (0,8)	3.596 (39,8)	80 (0,9)	1.723 (19,1)	2,59	1,48
17	Falta de conhecimento sobre as precauções-padrão	1.672 (18,5)	52 (0,6)	2.381 (26,3)	58 (0,6)	4.876 (53,9)	3,71	1,55
18	Rotina, hábitos e equipe de trabalho	2.903 (32,1)	57 (0,6)	3.723 (41,2)	95 (1,1)	2.261 (25,0)	2,86	1,51
19	Crenças pessoais relacionadas às precauções-padrão	3.725 (41,2)	75 (0,8)	2.768 (30,6)	55 (0,6)	2.416 (26,7)	2,71	1,63
**Item da escala**		**Nunca** **n (%)**	**Raramente** **n (%)**	**Às vezes** **n (%)**	**Muitas vezes** **n (%)**	**Sempre** **n (%)**	**Média**	**Desvio padrão**
20	Problemas relacionados ao material (qualidade, disponibilidade e acessibilidade)	1.278 (14,1)	50 (0,6)	2.823 (31,2)	86 (1,0)	4.802 (53,1)	3,78	1,45
21	Mesmo quando o paciente for difícil	103 (1,1)	78 (0,9)	353 (3,9)	1.281 (14,2)	7.224 (79,9)	4,71	0,69
22	Mesmo quando houver pouco tempo	108 (1,2)	145 (1,6)	657 (7,3)	1.873 (20,7)	6.256 (69,2)	4,55	0,80
23	Mesmo quando minhas mãos estiverem doloridas ou machucadas	207 (2,3)	219 (2,4)	728 (8,1)	1.653 (18,3)	6.232 (68,9)	4,49	0,91
24	Mesmo em uma situação de urgência	125 (1,4)	208 (2,3)	818 (9,0)	1.979 (21,9)	5.909 (65,4)	4,48	0,86

* *Missing.*

A escala SPQ apresentou uma média geral de 3,91 (DP= 0,48). Quanto aos domínios da escala, o fator organização apresentou maior pontuação (4,69; DP: 0,68), seguido da intenção de seguir as PP (4,56; DP: 0,70) (Tabela 3).

**Tabela 3:** Escore de determinantes sociocognitivos referente aos fatores da versão brasileira do *Standard Precautions Questionnaire* dos profissionais de enfermagem (N=9.039), Brasil, 2020

Fatores	Média	Desvio padrão
Atitudes	4,19	0,81
Influência social	3,74	1,06
Organização	4,69	0,68
Comportamento interpessoal	4,28	1,01
Restrições organizacionais	3,03	1,19
Restrições individuais	3,27	1,16
Intenção de seguir as precauções-padrão	4,56	0,70

Na análise dos escores dos fatores do SPQ-PB segundo a categoria profissional (Tabela 4), os enfermeiros obtiveram efeito significativo nas pontuações de organização (enfermeiro: 4,72/auxiliar ou técnico de enfermagem: 4,63; p<0,01), restrições individuais (enfermeiro: 3,35/auxiliar ou técnico de enfermagem: 3,11; p<0,01) e intenção de seguir as precauções-padrão (enfermeiro: 4,58/auxiliar ou técnico de enfermagem: 4,52; p<0,01). Enquanto isso, técnicos e auxiliares pontuaram mais nos demais fatores, como atitudes (auxiliar ou técnico de enfermagem: 4,24/enfermeiro: 4,17; p<0,01), influência social (auxiliar ou técnico de enfermagem: 3,99/enfermeiro: 3,60; p<0,01) e comportamento interpessoal (auxiliar ou técnico de enfermagem: 4,39/enfermeiro: 4,22; p<0,01). Para o fator restrições organizacionais, não houve diferenças significativas entre as categorias (p = 0,250).

**Tabela 4:** Escore de determinantes sociocognitivos referente aos fatores da versão brasileira do *Standard Precautions Questionnaire* por categoria profissional dos profissionais de enfermagem (N=9.039), Brasil, 2020

Fatores	Categoria profissional		
	Enfermeiro	Auxiliar ou técnico de enfermagem	Valor de *p* ^*^
Atitudes	4,17	4,24	< 0,01
Influência social	3,60	3,99	< 0,01
Organização	4,72	4,63	< 0,01
Comportamento interpessoal	4,22	4,39	< 0,01
Restrições organizacionais	3,04	3,00	0,250
Restrições individuais	3,35	3,11	< 0,01
Intenção de seguir as precauções-padrão	4,58	4,52	< 0,01

* *Teste de Mann-Whitney.*

## DISCUSSÃO

O presente estudo avaliou os fatores sociocognitivos determinantes na adesão às PP pelos profissionais de enfermagem na prática assistencial na pandemia de COVID-19 no Brasil. Os participantes reconheceram as PP como medidas eficazes para reduzir infecções e relataram intenção de segui-las.

A percepção da eficácia, o conhecimento das medidas preventivas, bem como a intenção de segui-las são fundamentais, visto que se trata de um fator individual que interfere diretamente no processo de adesão ou não dos trabalhadores da saúde às PP^(12)^, ao passo que implicam a percepção pessoal do risco e, consequentemente, a extensão da proteção adotada e o comportamento de risco assumido. Portanto, profissionais que reconhecem seu ambiente laboral como de baixo ou médio risco têm maiores chances de se acidentar, quando comparados com aqueles que avaliam o risco como alto, isso porque a adesão do EPI está intimamente relacionada à percepção que os profissionais têm acerca dos riscos a que estão expostos^(17)^.

Estudo realizado no Centro-Oeste do Brasil constatou que o conhecimento da equipe de enfermagem sobre as PP era inferior ao recomendado. Ainda, evidenciou que fatores individuais, relativos ao trabalho, incluindo a percepção de risco e de obstáculos para seguir as PP, além dos organizacionais, como treinamento e disponibilidade de EPI, impactam a adesão às PP^(18)^. Tais dados convergem com os achados da presente pesquisa, em que, nos domínios de restrições organizacionais e restrições individuais, foram indicados como fatores dificultadores problemas relacionados ao material, incluindo qualidade, disponibilidade e acessibilidade de EPI, seguidos da falta de conhecimento sobre as PP, mencionada anteriormente, e carga de trabalho mais elevada.

O conhecimento insatisfatório pode impactar baixos índices de adesão e podem ter correlação com lacunas na formação profissional, além da autoconfiança decorrente dos anos de experiência profissional, a lacuna entre o conhecimento e a prática, e desatualização quanto às pesquisas atuais, dificultando as bases de conhecimento sólidas para prevenção e controle de infecções. Ainda, jornadas de trabalhos elevadas podem desencadear estresse e fadiga, que podem afetar os processos de cognição do profissional, com consequente prejuízo às aplicações das medidas de segurança^(3,19)^, como as PP.

No que se refere ao domínio organização, a capacitação quanto às PP foi destacada como facilitadora da adesão. Assim, ressalta-se a necessidade da capacitação, através de atividades de educação em saúde com enfoque na adesão às PP e seus fatores intervenientes, por se tratar de temática complexa, dinâmica, multifacetada e de responsabilidade organizacional, que possibilita a sensibilização e conscientização do profissional quanto à relevância da adesão às PP^(18-19)^. Destaca-se que os profissionais apresentam uma maior adesão logo após os treinamentos realizados pela instituição de saúde, entretanto as medidas são descontinuadas com o passar do tempo. Portanto, observa-se a relevância de estratégias educacionais continuadas e permanentes, além de treinamentos constantes para a efetividade da ação^(3,19)^.

Entre os principais motivos para baixa adesão às PP e de contato, revisão apontou os fatores individuais, deficiências na formação, estrutura organizacional, problemas relacionados ao gerenciamento da unidade e as condições de trabalho^(3)^. Desse modo, a gestão institucional é fundamental para que a participação da equipe nessas atividades e treinamentos ocorra de forma contínua, através de um ambiente de cuidado favorável à adesão, com EPI em qualidade e quantidade suficiente, de fácil acesso, para facilitar e favorecer o seu uso, além de um clima de segurança institucional^(18)^, propiciado por uma estrutura física e supervisão de qualidade, aliadas à padronização e à socialização de rotinas^(12)^. Um ambiente inseguro implica a redução da motivação e interesse do profissional, predispondo-se a erros e agravos^(20)^.

No que se refere ao ambiente de trabalho/influência social, reconhecem que correm o risco de receber advertências dos seus superiores se não seguir as PP, o que demonstra uma preocupação da gestão quanto à temática e suas implicações para o profissional e pacientes. Isso reflete uma percepção institucional positiva, desde que seja propiciado um ambiente de cuidado favorável para aplicação dessas medidas, como estrutura física e de materiais de qualidade, conforme mencionado anteriormente.

A convivência constante com a exposição ao material biológico, que impacta a falta do medo de se contaminar, e a crença de que nada irá acontecer, elevando o sentimento da autoconfiança do trabalhador, favorecem a redução da percepção dos riscos pelos profissionais, um dos fatores (individuais) mais complexos, influenciando o comportamento dos profissionais e dificultando a tomada de decisão para a adoção das PP^(12)^. Portanto, ter um colega de trabalho adotando as medidas preventivas de forma correta favorece a percepção do ambiente de risco e maior adesão às PP, ao passo que, em ambientes onde as relações interpessoais não são saudáveis, impacta a falta de motivação do uso. Logo, colegas de trabalho podem influenciar de forma positiva ou negativa^(20)^.

Destarte, a adesão aos EPI é determinada pelo contexto vivenciado, no ambiente de trabalho, e por valores e crenças individuais, mas a decisão sobre o uso em geral é individual. Os fatores individuais, como desconforto, incômodo, descuido, esquecimento, falta de hábito e descrença, podem ser amplamente agravados pelos institucionais/organizacionais, como precária infraestrutura, falta de EPI suficiente e de qualidade, falta de conhecimento devido à não existência de educação permanente e sobrecarga de trabalho, que implicam desgaste físico e mental, impactando a cognição^(20)^. Desse modo, reconhecer os fatores individuais e institucionais é de suma importância, visando propiciar bases que minimizem as barreiras, enfraquecem a percepção da susceptibilidade e da severidade ao risco e reduzem a adesão às PP, favorecendo os facilitadores.

Estudo que aplicou a SPQ-PB com 300 profissionais de saúde evidenciou que os enfermeiros obtiveram efeito significativo nas pontuações de intenção (4,77; p=0,000) e restrições individuais (3,52; p=0,041)^(16)^, semelhante à presente pesquisa.

### Limitações do estudo

A pesquisa apresenta como limitação a possibilidade de uma super-representação de profissionais com habilidade em relação ao uso de computadores e redes sociais, tendo em vista o seu desenvolvimento *online*. Contudo, diante do número elevado de participantes, tais limitação não interferiram de forma significativa nos resultados.

### Contribuições para as áreas da enfermagem, saúde, ou políticas públicas

Os resultados podem contribuir para uma maior compreensão dos fatores determinantes na adesão às PP e, assim, possibilitar novas estratégias interventivas para redução dos fatores dificultadores e promoção dos facilitadores na prática laboral desses profissionais, incluindo a priorização desta temática nos programas de capacitação, elaboração e/ou reformulação de políticas públicas e protocolos institucionais, visando, assim, a redução de infecções cruzadas e riscos ocupacionais ocasionados pela baixa adesão ou uso errôneo das PP, com consequentes prejuízos institucionais.

## CONCLUSÕES

Entre os fatores determinantes na adesão às PP pelos profissionais de enfermagem na prática assistencial na pandemia de COVID-19 no Brasil, o fator organização apresentou maior pontuação, logo, maior impacto na adesão, seguido da intenção de seguir às PP. Além disso, os participantes reconheceram as PP como medidas eficazes para reduzir as infecções e sinalizaram a capacitação como facilitador da adesão às PP e problemas relacionados ao material como principal dificultador.

Diante de tais achados, possibilitou-se o desenvolvimento de estratégias interventivas, capacitações, treinamentos e educação continuada que foquem nos principais fatores determinantes na adesão às PP, além do direcionamento de estratégias de melhoria do cumprimento delas, para, assim, fortalecer a segurança do paciente, reduzir a exposição e riscos ocupacionais entre profissionais de saúde, bem como a ocorrência de acidentes laborais.
